# Urinary polyaromatic hydrocarbons are associated with adult emphysema, chronic bronchitis, asthma, and infections: US NHANES, 2011–2012

**DOI:** 10.1007/s11356-016-7867-7

**Published:** 2016-10-24

**Authors:** Ivy Shiue

**Affiliations:** 1 Faculty of Health and Life Sciences, Northumbria University, Newcastle upon Tyne, England NE1 8ST UK; 2 Owens Institute for Behavioral Research, University of Georgia, Athens, GA USA

**Keywords:** Polyaromatic hydrocarbons, Risk factor, Asthma, Chronic bronchitis, Emphysema, Infection, Chemicals

## Abstract

Links between environmental chemicals and human health have emerged over the last few decades, but the effects from polyaromatic hydrocarbons were less studied, compared to other commonly known environmental chemicals such as heavy metals, phthalates, arsenic, phenols, pesticides, etc. Therefore, the aim of the study was to examine the relationships of urinary polyaromatic hydrocarbons and adult respiratory health conditions using a large human sample in a national and population-based setting in recent years. Data were retrieved from United States National Health and Nutrition Examination Surveys, 2011–2012 including demographics, self-reported health conditions, and urinary polyaromatic hydrocarbons. Statistical analyses including chi-square test, *t* test, and survey-weighted logistic regression modeling were performed. Of 5560 American adults aged 20–80, urinary 2-hydroxyfluorene and 3-hydroxyfluorene were positively associated with emphysema (OR, 1.60, 95 % CI 1.26 to 2.03, *P* = 0.001 and OR, 1.42, 95 % CI 1.15 to 1.77, *P* = 0.003, respectively) and chronic bronchitis (OR, 1.42, 95 % CI 1.04 to 1.94, *P* = 0.031 and OR, 1.40, 95 % CI 1.03 to 1.91, *P* = 0.036, respectively), while 2-hydroxynaphthalene (2-naphthol) was likely to be borderline associated with emphysema and chronic bronchitis. Conversely, urinary 1-hydroxyphenanthrene, 3-hydroxyphenanthrene, 1-hydroxypyrene, and 4-hydroxyphenanthrene were inversely associated with asthma and infections. Urinary polyaromatic hydrocarbons are associated with adult respiratory health conditions, although the causality cannot be established. For future research, studies using large human sample across regions to longitudinally monitor would be suggested. For practice and policy-making, regulation on minimizing polyaromatic hydrocarbons exposure to protect respiratory health might need to be considered in future health and environmental policies and intervention programs.

## Introduction

### Evidence before this study

Links between environmental chemicals and human health including self-rated health, hypertension, cardiovascular disease, food allergy, oral health, emotional support, and cognitive function in American adults have emerged in Americans (Shiue 2015a; Shiue 2015b; Shiue 2015c; Shiue 2015d; Shiue 2015e; Shiue 2014; Shiue 2013a; Shiue 2013b; Shiue 2013c), but the effects from polyaromatic hydrocarbons (PAHs) were less studied, compared to other commonly known environmental chemicals such as heavy metals, arsenic, phenols, phthalates, etc. PAHs constitute a group of chemicals that people could be exposed via vehicle exhausts, asphalt, coal tar, wild fires, agricultural burning, soil, charbroiled foods, and tobacco smoke. Approximately, everyone could be exposed to PAHs on a daily basis from multiple sources. PAH pollution may have significant health implications, and the extent of damage to organisms from PAH exposure could be dependent on several factors including degrees and types of PAH exposure (Ball and Truskewycz 2013).

### Knowledge gap

Previously, animal models under a laboratory condition using rodents showed that exposure to PAHs adversely affected immunologic health (Luebke et al. 1997). However, research in this topic from human sample has not been well conducted. Providing evidence using human sample might help environmental health promotion in the next few years. Recently, associations of PAHs and cardiovascular, oral, emotional, and self-rated health have been observed (Shiue 2015a; Shiue 2015b; Shiue 2015c; Shiue 2015d; Shiue 2015e), but those on respiratory health have not been documented.

### Study aim

Following this context, therefore, the aim of the present study was to examine the relationships of urinary PAHs and adult respiratory conditions using a large human sample in a national and population-based setting in recent years.

## Methods

### Study sample

As described elsewhere (Centers for Disease Control and Prevention 2012), United States National Health and Nutrition Examination Surveys (NHANES) has been a national, population-based, multi-year, cross-sectional study. Study samples are representative sample of the civilian, non-institutionalized US population. Information on demographics (more details via http://wwwn.cdc.gov/nchs/nhanes/2011-2012/DEMO_G.htm), serum cotinine (more details via http://wwwn.cdc.gov/nchs/nhanes/2011-2012/COTNAL_G.htm), and self-reported respiratory health conditions (more details via http://wwwn.cdc.gov/nchs/nhanes/2011-2012/MCQ_G.htm) was obtained by household interview using questionnaires. In the current analysis, the 2011–2012 study cohort as the most recent wave with data on urinary PAHs was selected. Informed consents were obtained from participating subjects by the NHANES researchers.

### Biomonitoring

Urines were only collected in a subsample, being one third of the whole study cohort with representation (more details via http://www.cdc.gov/nchs/data/nhanes/nhanes_09_10/homeurine.pdf), to measure environmental chemical concentrations in urines among people aged 6 and above (more details via http://www.cdc.gov/nchs/nhanes/nhanes2011-2012/labdoc_g.htm). Urine specimens from urinary polyaromatic hydrocarbon were processed, stored under appropriate frozen (−20 °C) conditions, and shipped to the Division of Environmental Health Laboratory Sciences, National Center for Environmental Health, Centers for Disease Control and Prevention for analysis. According to the NHANES website (more details via http://www.cdc.gov/nchs/data/nhanes/nhanes_11_12/PAH_G_met.pdf), the procedure involved enzymatic hydrolysis of glucuronidated/sulfated OH-polyaromatic hydrocarbon metabolites in urine, extraction, derivatization, and analysis using isotope dilution capillary gas chromatography tandem mass spectrometry (GC-MS/MS). Ion transitions specific to each analyte and carbon-13-labeled internal standards are monitored, and the abundances of each ion are measured. Since urinary hydrocarbon concentrations were highly skewed, they were all log transformed when carrying out the statistical analyses.

### Variables and analysis

Adults aged 20 and above were included in the current statistical analysis since chronic diseases were commonly reported in adults. Associations of urinary PAHs and adult self-reported respiratory health conditions were examined by using *t* test and survey-weighted logistic regression model, presenting with mean values, odds ratios (OR), and 95 % confidence intervals (CI). Covariates including urinary creatinine, age, sex, ratio of family income to poverty (proxy of socioeconomic status), body mass index, education level, serum cotinine (biomarker of smoking status), alcohol status, and physical activity level were adjusted. Statistical software STATA version 13.0 (STATA, College Station, Texas, USA) was used to perform all the statistical analyses.

### Ethics consideration

Since there is only secondary data analyses employed without any participant personal information identified by extracting statistical data from the NHANES website in the present study, no further ethics approval for conducting the present study is required (more details via http://www.ethicsguidebook.ac.uk/Secondary-analysis-106).

## Results

### Descriptive statistics

Of 5560 American adults aged 20–80 and included in the statistical analysis, their characteristics are shown in Table 1. The presence of different respiratory health conditions in the American adult population varied and is accordingly presented in Table 2.

**Table 1:** Characteristics of the included participants aged 20–80 (*n* = 5560)

	*N* (%)
Age	48.9 ± 17.9
20–39	1957 (35.2 %)
40–59	1812 (32.6 %)
60–80	1791 (32.2 %)
Sex	
Male	2740 (49.3 %)
Female	2820 (50.7 %)
Body mass index	28.8 ± 6.9
<18.5	1103 (1.9 %)
18.5–24.9	1577 (28.4 %)
25–29.9	1684 (30.3 %)
30+	2196 (39.5 %)
Ratio of family income to poverty	
0–4.9	4199 (75.5 %)
5+	1361 (24.5 %)
Education level	
Less than 9th grade	550 (9.9 %)
9–11th grade	782 (14.1 %)
High school graduate or equivalent	1169 (21.0 %)
Some college or AA degree	1657 (29.8 %)
College graduate or above	1397 (25.2 %)
Serum cotinine (ng/mL)	52.1 ± 120.2
Alcohol status	
>12 drinks	3413 (72.8 %)
Less than 12 drinks	1275 (27.2 %)
Physical activity level	
Engaging moderately	2297 (41.3 %)
None	3262 (58.7 %)

**Table 2:** Prevalence of respiratory health conditions in American adults (*n* = 5560)

	*N* (%)
Asthma	810 (14.6 %)
Emphysema	100 (1.8 %)
Chronic bronchitis	297 (5.4 %)
Wheezing	749 (13.5 %)
Coughing	376 (10.4 %)
Hay fever	879 (15.9 %)
Infections	1015 (28.4 %)

### Analytical statistics

In Tables 3, 4, 5, 6, 7, 8, 9, 10, 11, and 12, associations of 10 urinary PAHs and adult respiratory health conditions are listed separately. In general, urinary PAHs were higher in people with emphysema or chronic bronchitis but lower in people with asthma or infections. No associations were found between urinary PAHs and wheezing, coughing, and hay fever. Specifically, urinary 2-hydroxyfluorene and 3-hydroxyfluorene were positively associated with emphysema (OR, 1.60, 95 % CI 1.26 to 2.03, *P* = 0.001 and OR, 1.42, 95 % CI 1.15 to 1.77, *P* = 0.003, respectively) and chronic bronchitis (OR, 1.42, 95 % CI 1.04 to 1.94, *P* = 0.031 and OR, 1.40, 95 % CI 1.03 to 1.91, *P* = 0.036, respectively), while 2-hydroxynaphthalene (2-naphthol) was likely to be borderline associated with emphysema (OR, 1.20, 95 % CI 0.82 to 1.75, *P* = 0.332) and chronic bronchitis (OR, 1.32, 95 % CI 1.02 to 1.72, *P* = 0.038). Conversely, urinary 1-hydroxyphenanthrene, 3-hydroxyphenanthrene, 1-hydroxypyrene, and 4-hydroxyphenanthrene were inversely associated with asthma and infections.

**Table 3:** Associations between 2-hydroxyfluorene (ng/L) and adult health (*n* = 1670)

	Present	Absent	*P* value	OR (95 % CI)^a^	*P* value
Asthma	702.3 (1094.0)	585.0 (1056.2)	0.112	0.86 (0.70–1.06)	0.144
Emphysema	1174.7 (1678.9)	591.5 (1047.3)	0.005	1.60 (1.26–2.03)	0.001
Chronic bronchitis	1006.9 (1322.1)	578.3 (1042.1)	0.0002	1.42 (1.04–1.94)	0.031
Wheezing	880.6 (1210.7)	561.0 (1032.9)	<0.001	1.20 (0.89–1.62)	0.216
Coughing	1148.2 (1894.5)	523.6 (963.2)	<0.001	1.14 (0.77–1.68)	0.484
Hay fever	446.7 (692.0)	631.6 (1120.2)	0.007	0.80 (0.73–1.06)	0.165
Infections	547.8 (918.5)	611.7 (1194.3)	0.392	0.89 (0.76–1.04)	0.140

^a^Adjusted for urine creatinine, age, sex, body mass index, ratio of family income to poverty, education level, serum cotinine, alcohol habit, physical activity level, and subsampling weighting

**Table 4:** Associations between 3-hydroxyfluorene (ng/L) and adult health (*n* = 1670)

	Present	Absent	*P* value	OR (95 % CI)^a^	*P* value
Asthma	341.9 (663.2)	288.1 (605.5)	0.209	0.88 (0.73–1.05)	0.152
Emphysema	600.1 (1004.8)	290.6 (605.0)	0.009	1.42 (1.15–1.77)	0.003
Chronic bronchitis	524.7 (805.6)	282.5 (599.5)	0.0003	1.40 (1.03–1.91)	0.036
Wheezing	451.8 (680.3)	273.0 (600.6)	0.0001	1.23 (0.93–1.62)	0.140
Coughing	540.9 (794.6)	247.2 (563.4)	<0.001	1.15 (0.83–1.59)	0.369
Hay fever	216.6 (404.9)	310.9 (646.5)	0.018	0.91 (0.75–1.11)	0.319
Infections	249.3 (466.2)	292.4 (649.4)	0.283	0.89 (0.76–1.04)	0.145

^a^Adjusted for urine creatinine, age, sex, body mass index, ratio of family income to poverty, education level, serum cotinine, alcohol habit, physical activity level, and subsampling weighting

**Table 5:** Associations between 9-hydroxyfluorene (ng/L) and adult health (*n* = 1670)

	Present	Absent	*P* value	OR (95 % CI)^a^	*P* value
Asthma	515.1 (736.7)	519.9 (888.3)	0.937	0.76 (0.64–0.90)	0.003
Emphysema	576.5 (613.8)	518.0 (871.9)	0.729	1.29 (0.87–1.91)	0.195
Chronic bronchitis	678.0 (941.5)	509.8 (864.0)	0.079	1.17 (0.84–1.65)	0.328
Wheezing	615.7 (829.7)	504.9 (872.9)	0.080	1.04 (0.75–1.43)	0.812
Coughing	764.4 (999.1)	486.6 (700.8)	0.0001	1.15 (0.74–1.77)	0.511
Hay fever	439.0 (615.0)	533.3 (910.6)	0.095	0.95 (0.81–1.10)	0.447
Infections	476.5 (629.8)	535.8 (788.5)	0.232	0.85 (0.70–1.04)	0.109

^a^Adjusted for urine creatinine, age, sex, body mass index, ratio of family income to poverty, education level, serum cotinine, alcohol habit, physical activity level, and subsampling weighting

**Table 6:** Associations between 1-hydroxyphenanthrene (ng/L) and adult health (*n* = 1670)

	Present	Absent	*P* value	OR (95 % CI)^a^	*P* value
Asthma	196.5 (216.6)	202.8 (295.5)	0.749	0.68 (0.55–0.83)	0.001
Emphysema	206.6 (239.7)	201.7 (286.4)	0.930	1.03 (0.57–1.87)	0.918
Chronic bronchitis	218.4 (206.5)	200.7 (289.4)	0.575	0.97 (0.61–1.55)	0.883
Wheezing	203.4 (202.3)	201.6 (295.7)	0.930	0.87 (0.70–1.09)	0.214
Coughing	269.6 (708.8)	193.2 (230.0)	0.015	0.99 (0.66–1.47)	0.953
Hay fever	178.8 (211.3)	206.0 (298.3)	0.142	0.91 (0.78–1.06)	0.205
Infections	176.8 (176.5)	213.0 (362.0)	0.088	0.78 (0.64–0.96)	0.019

^a^Adjusted for urine creatinine, age, sex, body mass index, ratio of family income to poverty, education level, serum cotinine, alcohol habit, physical activity level, and subsampling weighting

**Table 7:** Associations between 2-hydroxyphenanthrene (ng/L) and adult health (*n* = 1670)

	Present	Absent	*P* value	OR (95 % CI)^a^	*P* value
Asthma	111.3 (123.0)	108.3 (156.5)	0.780	0.83 (0.67–1.02)	0.067
Emphysema	117.5 (111.8)	108.5 (152.8)	0.760	1.42 (0.96–2.09)	0.077
Chronic bronchitis	123.7 (105.8)	107.9 (154.3)	0.351	1.24 (0.76–2.02)	0.359
Wheezing	113.2 (105.8)	108.0 (157.7)	0.640	1.04 (0.79–1.38)	0.766
Coughing	143.1 (313.8)	101.3 (121.1)	0.006	1.01 (0.66–1.55)	0.961
Hay fever	94.7 (113.8)	111.4 (158.8)	0.093	0.95 (0.78–1.17)	0.630
Infections	90.2 (88.2)	112.8 (174.2)	0.029	0.81 (0.64–1.04)	0.091

^a^Adjusted for urine creatinine, age, sex, body mass index, ratio of family income to poverty, education level, serum cotinine, alcohol habit, physical activity level, and subsampling weighting

**Table 8:** Associations between 3-hydroxyphenanthrene (ng/L) and adult health (*n* = 1670)

	Present	Absent	*P* value	OR (95 % CI)^a^	*P* value
Asthma	122.8 (160.7)	122.6 (237.8)	0.991	0.77 (0.63–0.93)	0.008
Emphysema	121.1 (150.5)	122.6 (229.6)	0.973	0.97 (0.60–1.58)	0.902
Chronic bronchitis	144.2 (179.4)	121.2 (230.8)	0.364	1.12 (0.77–1.64)	0.529
Wheezing	129.6 (150.0)	121.6 (237.6)	0.629	1.06 (0.80–1.39)	0.670
Coughing	191.3 (624.9)	109.1 (149.2)	0.001	1.06 (0.73–1.55)	0.742
Hay fever	100.5 (136.0)	126.9 (242.9)	0.075	0.89 (0.76–1.04)	0.124
Infections	93.0 (101.3)	129.1 (289.7)	0.030	0.76 (0.63–0.92)	0.008

^a^Adjusted for urine creatinine, age, sex, body mass index, ratio of family income to poverty, education level, serum cotinine, alcohol habit, physical activity level, and subsampling weighting

**Table 9:** Associations between 1-hydroxypyrene (ng/L) and adult health (*n* = 1670)

	Present	Absent	*P* value	OR (95 % CI)^a^	*P* value
Asthma	217.2 (306.8)	200.7 (333.0)	0.470	0.79 (0.65–0.96)	0.019
Emphysema	229.5 (377.8)	202.5 (328.7)	0.673	1.07 (0.70–1.62)	0.740
Chronic bronchitis	248.7 (310.1)	200.2 (330.4)	0.181	1.30 (0.91–1.87)	0.140
Wheezing	239.2 (328.5)	197.7 (329.2)	0.084	1.05 (0.80–1.37)	0.727
Coughing	280.5 (609.3)	175.0 (288.1)	0.002	0.99 (0.70–1.42)	0.968
Hay fever	176.8 (282.0)	207.8 (336.9)	0.147	0.84 (0.71–1.00)	0.046
Infections	168.4 (274.4)	194.9 (363.3)	0.241	0.83 (0.72–0.97)	0.020

^a^Adjusted for urine creatinine, age, sex, body mass index, ratio of family income to poverty, education level, serum cotinine, alcohol habit, physical activity level, and subsampling weighting

**Table 10:** Associations between 1-hydroxynaphthalene (1-naphthol) (ng/L) and adult health (*n* = 1670)

	Present	Absent	*P* value	OR (95 % CI)^a^	*P* value
Asthma	65,790.7 (827,032.2)	32,590.7 (555,198.9)	0.427	0.93 (0.79–1.10)	0.377
Emphysema	13,920.9 (28,619.5)	37,656.7 (605,732.7)	0.839	1.07 (0.83–1.38)	0.576
Chronic bronchitis	170,804.4 (1,371,824.0)	30,136.6 (529,120.2)	0.034	1.06 (0.85–1.32)	0.572
Wheezing	76,277.2 (874,264.1)	31,635.6 (550,415.4)	0.309	1.01 (0.82–1.24)	0.945
Coughing	142,167.3 (1,196,975.0)	45,590.4 (682,159.8)	0.194	1.18 (0.94–1.49)	0.143
Hay fever	107,878.5 (1,239,498.0)	23,182.9 (354,243.4)	0.030	0.91 (0.74–1.12)	0.348
Infections	142,826.6 (1,345,310.0)	19,376.2 (205,077.5)	0.014	0.97 (0.79–1.19)	0.751

^a^Adjusted for urine creatinine, age, sex, body mass index, ratio of family income to poverty, education level, serum cotinine, alcohol habit, physical activity level, and subsampling weighting

**Table 11:** Associations between 2-hydroxynaphthalene (2-naphthol) (ng/L) and adult health (*n* = 1670)

	Present	Absent	*P* value	OR (95 % CI)^a^	*P* value
Asthma	9851.4 (11,745.6)	8756.0 (12,149.8)	0.193	0.88 (0.74–1.04)	0.134
Emphysema	11,964.7 (15,808.4)	8852.3 (12,026.0)	0.185	1.20 (0.82–1.75)	0.332
Chronic bronchitis	11,858.9 (13,145.8)	8732.6 (12,024.7)	0.019	1.32 (1.02–1.72)	0.038
Wheezing	12,355.6 (17,029.1)	8409.5 (11,124.2)	<0.001	1.09 (0.86–1.38)	0.462
Coughing	12,665.5 (21,306.8)	7687.7 (10,106.7)	<0.001	1.13 (0.78–1.65)	0.490
Hay fever	6621.9 (7993.0)	9359.7 (12,725.6)	0.001	0.86 (0.71–1.03)	0.097
Infections	8501.7 (11,200.4)	8136.1 (12,233.8)	0.646	0.96 (0.76–1.22)	0.714

^a^Adjusted for urine creatinine, age, sex, body mass index, ratio of family income to poverty, education level, serum cotinine, alcohol habit, physical activity level, and subsampling weighting

**Table 12:** Associations between 4-hydroxyphenanthrene (ng/L) and adult health (*n* = 1670)

	Present	Absent	*P* value	OR (95 % CI)^a^	*P* value
Asthma	42.4 (79.9)	33.9 (44.9)	0.017	0.80 (0.67–0.94)	0.012
Emphysema	35.5 (39.1)	35.1 (51.6)	0.964	1.05 (0.58–1.88)	0.872
Chronic bronchitis	41.5 (39.3)	34.7 (52.0)	0.233	1.18 (0.83–1.68)	0.327
Wheezing	37.7 (35.9)	34.7 (53.2)	0.419	1.06 (0.79–1.41)	0.681
Coughing	38.0 (41.8)	33.8 (55.2)	0.430	1.08 (0.66–1.78)	0.733
Hay fever	31.8 (51.0)	35.7 (51.5)	0.240	0.96 (0.81–1.14)	0.625
Infections	29.5 (32.4)	36.4 (60.9)	0.058	0.74 (0.58–0.94)	0.016

^a^Adjusted for urine creatinine, age, sex, body mass index, ratio of family income to poverty, education level, serum cotinine, alcohol habit, physical activity level, and subsampling weighting

## Discussion

### PAHs, emphysema, and chronic bronchitis

In animal studies, bitumen fumes or traffic exposure releasing hydrocarbons were observed to result in emphysema in rats (Gate et al. 2006; Wang et al. 1992). However, evidence from human sample was lacking. Consistent with the findings in the animal studies mentioned above, the present study has provided epidemiological evidence on the relationship of PAHs and emphysema from a large human sample. Moreover, the link between PAHs and adult chronic bronchitis has been continuously documented. Previous evidence was obtained in 598 Brazilian male workers (Mendonça et al. 2007), 211 Swedish loggers (Hagberg et al. 1985), and 138 Polish steel mill workers (Kolarzyk et al. 2000). Again, consistent with these studies, the present study has further provided epidemiological evidence drawn from the general population to report such association.

### PAHs, asthma, and infections

The link of PAHs and asthma has not been established and that of PAHs and infections has not been well documented as well. Similar to a previous study in 184 American volunteers showing urinary 1-hydroxypyrene was observed to be less in people with hepatitis virus infection (Johnson et al. 2010), the present study also presented the inverse associations of 1-hydroxyphenanthrene, 3-hydroxyphenanthrene, 1-hydroxypyrene, and 4-hydroxyphenanthrene and infections in the general population. However, the mechanism is unknown and would need longitudinal and/or experimental research to confirm or refute the finding.

### Strengths and limitations

The present study has a few strengths. Firstly, this study was conducted in a large and nationally representative human sample with mixed ethnicities and socioeconomic status. Secondly, this is the first time to examine the effects of urinary hydrocarbon concentrations on adult respiratory health conditions by symptoms. However, there are also a few limitations that cannot be ignored. First, there could be still other emerging chemicals from the living environments through different channels/vehicles that we might not yet know and would need future research to further identify and examine. Second, causality cannot be established in the present study due to the cross-sectional study design in nature. Taken together, future studies retaining the strengths and overcoming the limitations with a longitudinal and/or experimental study design to confirm or refute the current findings and, if at all, to understand the persisting effects along the life course from early years to old age would be recommended.

### Directions for future research, practice, and policy

In conclusion, urinary PAHs were positively associated with emphysema and chronic but inversely associated with asthma and infections. There were no associations between urinary PAHs and wheezing, coughing, and hay fever found. For future research, studies using large human sample across regions to longitudinally monitor would be suggested. For practice and policy-making, regulation on minimizing polyaromatic hydrocarbons exposure to protect respiratory health might need to be considered in future health and environmental policies and intervention programs.
